# Alleviation of Myocardial Inflammation in Diabetic Rats by Flavonoid Extract of Helichrysum Arenarium and Its Effect on Damaged Myocardial Cells Induced by High Glucose

**DOI:** 10.3389/fsurg.2022.873010

**Published:** 2022-04-14

**Authors:** Huanyu Liu, Wei Lan

**Affiliations:** College of Pharmacy, Xinjiang Medical University, Urumqi, China

**Keywords:** myocardial inflammation, diabetic, flavonoid extract of helichrysum arenarium, myocardial cells, high-glucose

## Abstract

**Objective:**

To investigate the effects of helichrysum arenarium flavonoid extract on high glucose damaged cardiomyocytes and the alleviation of myocardial inflammation in diabetic rats.

**Methods:**

The study was divided into two parts, the first part was a cellular experiment in which a high-glucose cardiomyocyte injury model (H9C2) was established using a high-glucose culture medium, divided into low (group N1, 6.25 μg/mL), medium (group N2, 12.5 μg/mL), high dose group (group N3, 25 μg/mL) of helichrysum arenarium intervention and a model control group. The levels of enzyme activities [creatine kinase (CK) and lactate dehydrogenase (LDH)] in each group of H9c2 cells were measured by Enzyme-linked immunosorbent assay (ELISA), the expression levels of apoptotic proteins (Bax and Bcl-2) by western blot (WB), and the expression levels of inflammatory factors [tumor necrosis factor-α (TNF-α), interleukin-1β (IL-1β), interleukin-6 (IL-6)] by RT-qPCR. The second part is animal experiments, after establishing the diabetic rat model, we used helichrysum arenarium flavonoid extract to intervene SD rats, divided into helichrysum arenarium intervention low (group S1, 250 mg/kg), medium (group S2, 500 mg/kg), high dose group (group S3, 1 g/kg), SD rat model group. Hematoxylin-eosin (HE) staining was used to observe myocardial tissue lesions, and Real Time Quantitative PCR (RT-qPCR) method was used to detect inflammatory (TNF-α, IL-1β, and IL-6) infiltration in myocardial tissue.

**Results:**

Cellular experiments: The activity levels of enzymes such as CK and LDH and the levels of inflammatory factors such as TNF-α, IL-1β, and IL-6 in damaged cardiac myocytes were significantly decreased after helichrysum arenarium intervention; the expression levels of Bax protein were significantly down-regulated and the expression levels of Bcl-2 protein expression were significantly up-regulated. Animal experiment: HE staining showed that the model group had widened intercellular spaces, interstitial edema and obvious inflammatory cell infiltration in cardiac muscle tissue. After the intervention of helichrysum arenarium, the collagen fibers of rat myocardial cells were significantly reduced and cell degeneration was alleviated. Animal experiment: HE staining showed that the model group had widened intercellular spaces, interstitial edema and obvious inflammatory cell infiltration in cardiac muscle tissue. After the intervention of helichrysum arenarium, the collagen fibers of rat myocardial cells were significantly reduced and cell degeneration was alleviated; the levels of TNF-α, IL-1β, IL-6 and other inflammatory factors in myocardial tissues were significantly decreased.

**Conclusion:**

The helichrysum arenarium flavonoid extract can reduce the degree of damage of H9C2 cells induced by high glucose and decrease the cellular inflammatory response, and its mechanism of action may be achieved by regulating the apoptotic factors Bax and Bcl-2. In addition, the extract of helichrysum arenarium can reduce the histopathological damage of myocardium in diabetic rats, decrease the inflammatory response in the tissue, and achieve the effect of myocardial protection.

## Introduction

With the change of people's lifestyle and diet structure, the progress of population aging and the increase of obesity rate, the global incidence of diabetes mellitus (DM) is surging at an alarming rate, and the prevention and treatment of DM has become a worldwide problem (1, 2). DM and its complications pose a serious threat to human health, especially cardiovascular complications, and an epidemiological follow-up survey (3) of DM patients found that the number of deaths due to cardiovascular diseases in the DM population was much higher than in the non-DM population. This phenomenon may be due to the fact that diabetes increases the incidence of cardiovascular disease by promoting cardiovascular atherosclerosis, and that the “glucotoxicity” and “lipotoxicity” of diabetes affects the function and normal physiological state of cardiomyocytes, eventually leading to heart failure and death (4, 5).

Diabetic cardiomyopathy (DCM) is a widespread focal myocardial necrosis caused by myocardial metabolic disorders and cardiac microangiopathy due to diabetes mellitus, and its pathological changes are mainly characterized by cardiomyocyte hypertrophy, myocardial fibrosis and myocardial collagen formation (6, 7). The specific mechanism of how DCM occurs is not clear, and many domestic and foreign scholars (8–10) believe that it may be due to the involvement of increased oxidative stress, abnormal myocardial energy metabolism, and activation of inflammatory pathways, causing apoptosis, necrosis, fibrosis, and other changes in myocardial cells, which ultimately lead to structural and functional changes in the myocardium.

At present, the treatment methods of DCM include traditional Chinese medicine treatment and western medicine treatment. Traditional western medicine treatment for DCM mainly uses hypoglycemic agents, insulin and insulin-producing agents, and β-blockers, all of which have unavoidable adverse effects and are prone to drug dependence (11, 12). Single herbs such as Milkvetch Root and Dan-Shen Root and herbal compounds such as Shengmai Powder and Decoction GuiQi, which are commonly used in Chinese medicine treatment, also have limitations due to the uncertainty of dosing and onset of action (13). While some herbs and their extracts have been reported as potential therapeutic agents for DCM in recent years, they have not yet entered the market in the form of highly effective drugs and need to be further studied in depth.

Helichrysum arenarium is a folkloric medicinal herb of Xinjiang with complex chemical composition, mainly divided into flavonoids, total saponins and phenolic acids. Reports by MORIKAWA (14), Kramberger (15) showed that helichrysum arenarium extract has hypoglycemic effect and in addition MORIKAWA found that its extract inhibited dipeptidyl peptidase-activity. In order to investigate more deeply the hypoglycemic effect of helichrysum arenarium extract, this study combined with the pharmacology of helichrysum arenarium network to improve DCM as an entry point, and to investigate the protective effect of helichrysum arenarium on high glucose-induced damaged cardiomyocytes and the alleviation of myocardial inflammation in diabetic rats.

## Materials and Methods

### Materials

#### Main Drugs, Reagents and Instruments

Helichrysum Arenarium was collected in April 2020 in the Altai region of Xinjiang, Streptozotocin (STZ) dry powder, RIPA lysis buffer, Paraformaldehyde solution (Sigma, USA); carbon dioxide thermostat culture (Thermo, USA); 96-well cell culture plate, DMEM/F-12 culture solution (GIBCO, USA); fetal bovine serum (Hyclone); fluorescent quantitative PCR Mix, reverse transcription kit, trizol reagents (Invitrogen, USA); pathology microtome (Leica, Germany), enzyme-labeled instrument (Bio-Tek, Germany); and fetal bovine serum (Hyclone). Rabbit Anti-Mouse TNF-α Antibody, Rabbit Anti-Mouse IL-1β Antibody, Rabbit Anti-Mouse IL-6 Antibody, Rabbit Anti-Mouse GAPDH Antibody (Bio-Tek, Beijing, China); Bax antibody, Bcl-2 antibody (Bio-Tek, Wuhan, China); CK ELISA kit, LDH ELISA kit (Yanjin Biological Company, Shanghai, China); Bicinchonic acid disodium salt (BCA) protein quantification kit (Bolf, Wuhan, China); blood glucose meter (Yi Cheng, Beijing, China).

#### Cell Grouping and Manipulation

Using trypsin digestion method, log phase H9c2 cells (purchased from the cell bank of Chinese Academy of Sciences) were made into cell suspensions, and each well was inoculated into 96-well plates at 5 × 10^4^, and 6 replicate wells were set up for each group, and incubated in a cell incubator (37°C, 5% CO_2_), and the cells were handled appropriately according to the grouping scheme after being attached to the wall. According to the purpose of the experiment, the cells were divided into four groups: model control group (HG group, high glucose stimulation at a concentration of 33.3 mmol/L for 48 h), low dose group (N1 group, high sugar concentration and treatment as model control, with 6.25 μg/mL of helichrysum arenarium extract added after handling), medium dose group (N2 group, high sugar concentration and treatment as model control, with 12.5 μg/mL of helichrysum arenarium extract added after handling), and high dose group (N3 group, high sugar concentration and treatment as model control, with 25 μg/mL of extracts of helichrysum arenarium was added after handling).

#### Animal Grouping and Handling

Healthy male SD rats weighing 180~200 g were selected and randomly divided into four groups, including low (S1 group, 250 mg/kg), medium (S2 group, 500 mg/kg), and high dose groups (S3 group, 1 g/kg) of helichrysum arenarium intervention and 4 groups of SD rat model (DCM group) after adaptive feeding for 1 week, 10 animals in each group. The DM model was constructed by intraperitoneal injection of STZ (30 mg/kg) in rats, and the DM modeling was considered successful for rats with blood glucose values higher than 16.7 mmol/L for 3 consecutive days. From day 4, rats in S1, S2, and S3 groups were given the corresponding concentrations of helichrysum arenarium extract by gavage for 8 weeks, while rats in DCM group were given the same volume of saline by gavage, and the drug doses were determined according to the pre-test and previous literature (16, 17). Body weight, blood glucose, blood lipids and fasting insulin were measured after 10 weeks of successful model establishment.

### Methods

#### Enzyme-Linked Immunosorbent Assay

The content of CK and LDH was determined according to the instructions of Shanghai Research and Technology Co. The steps were as follows: standard addition—sample addition— liquid preparation—washing—enzyme addition—incubation—washing—color development—termination of the reaction—determination of absorbance. The assay should be carried out within 15 min of the addition of the termination solution.

#### Protein Blotting (WB)

The cells were lysed using RIPA and quantified by disodium bis(quinolinic acid) (BCA) protein quantification kit at a uniform concentration of 100 μg. 20 μg was applied to each well, electrophoresis was performed, the membrane was transferred, 5% skimmed milk powder was closed at room temperature for 1.5 h, the membrane was washed with phosphate buffer saline (PBS-T), primary antibody (1:500), overnight at 4°C, rinsed, secondary antibody (1:5,000) at room temperature for 2 h, rinsed, and then luminescence developed in an electrochemiluminescence (ECL) imaging system. Glyceraldehyde-3phosphate dehydrogenase (GAPDH) was used as an internal reference, and the results were scanned in grayscale for semi-quantitative analysis.

#### Fluorescent Quantitative PCR (qRT-PCR)

Total mRNA was extracted from each group of H9c2 cells and rat myocardial tissue using TRIzol, and the total mRNA was reverse transcribed into cDNA and PCR amplified according to the instructions. PCR primers were designed as in Table 1 reverse transcription and qRT-PCR reactions were performed strictly according to the kit instructions. The relative expression of target genes was expressed as 2^−Δ*ΔCt*^.

**Table 1:** Target gene names and PCR primers.

**Name of gene**	**Primer sequences**	
TNF-α	Forward	5'-GCTGCACTTTGGAGTGATCG-3'
	Reverse	5'-GAGGGTTTGCTACAACATGGG-3'
IL-1β	Forward	5'-AGAAG- TACCTGAGCTCGCCA-3'
	Reverse	5'-CTG- GAAGGAGCACTTCATCTGT-3'
IL-6	Forward	5'-ACTCACCTCTTCAGAACGAATTG-3'
	Reverse	5'-CCATCTTTGGAAGGTTCAGGTTG-3'
GAPDH	Forward	5'-AGAAGGCTGGGGCTCATTTG-3'
	Reverse	5'-AGGGGCCATCCACAGTCTTC-3'

#### Hoechst (HE) Staining

Myocardial tissues (about 2 mm thick) were taken from rats, and myocardial tissues were fixed in 4% paraformaldehyde solution after washing with ice-cold saline, and then de-watered and paraffin-embedded after 48 h. The sections were stained according to HE staining kit, and the morphological and structural changes of myocardial cells were observed under the light microscope.

#### Blood Glucose and Lipid Testing

Blood glucose (GLU) level was measured by blood glucose meter; serum total cholesterol (TC) and triacylglycerol (TG) were measured by automatic biochemical analyzer, all operations were performed with reference to the kit instructions, and the relevant kits were provided by Beijing Ovia Biotechnology Co.

### Statistical Treatment

SPSS 21.0 software was used for statistical analysis. Statistical indicators were tested for normality and chi-square test, and each statistical data was expressed as mean ± standard deviation (Mean ± SD); one-way ANOVA was used for the analysis of multi-group measurement data, and the difference between two groups was analyzed by *t*-test. *p* < 0.05 indicated statistical differences. GraphPad Prism 8.0 software was used for statistical calculations and graphing.

## Results

### Comparison of Enzyme Activity Levels in H9c2 Cells in Each Group

**Figure 1:**
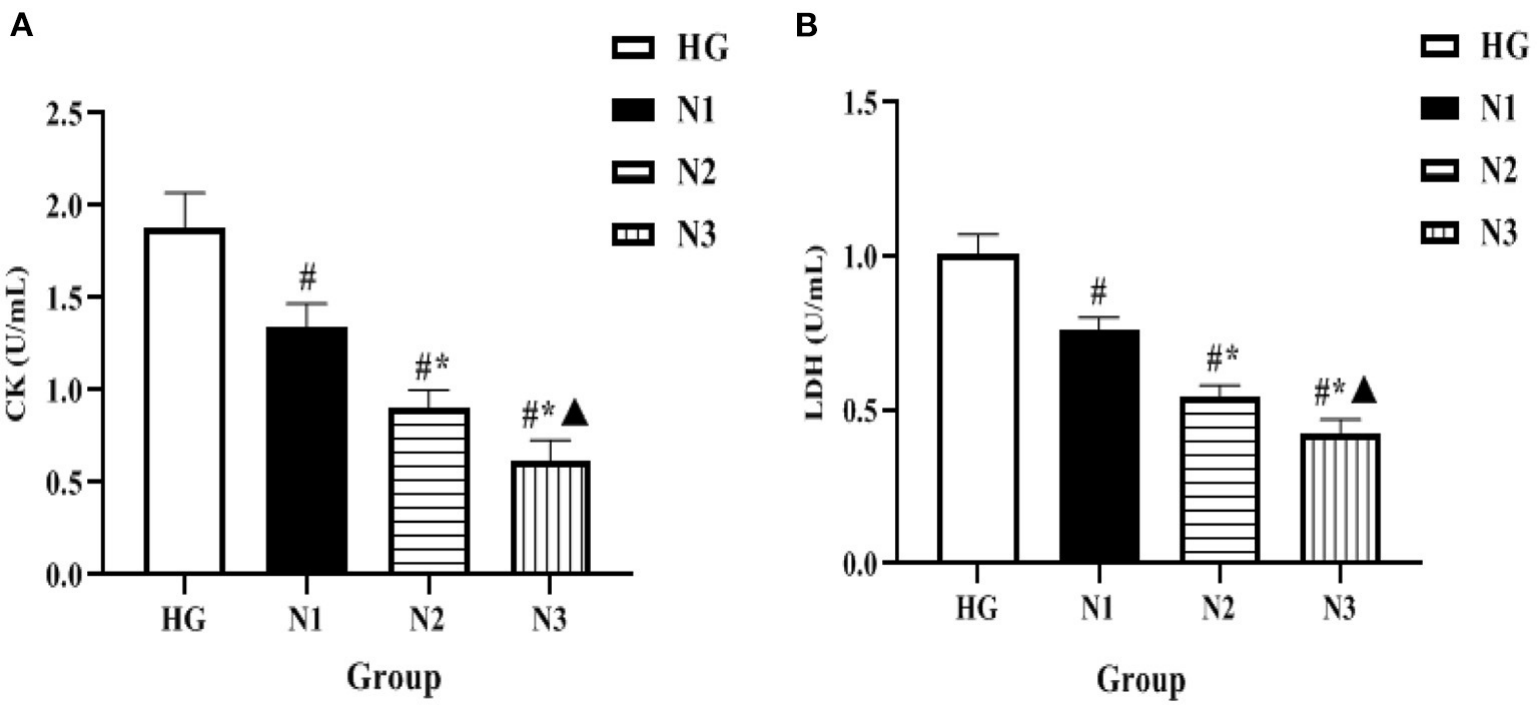
Comparison of enzyme activity levels in H9c2 cells in each group. In the above figure, **(A)** the comparison of CK levels in the four groups of H9c2 cells, and **(B)** the comparison of LDH levels in the four groups. Compared with HG group, ^#^*P* < 0.05; compared with N1 group, ^*^P < 0.05; compared with N2 group, ^▴^*P* < 0.05.

The levels of intracellular enzymes such as CK and LDH were significantly lower in the N1, N2, and N3 groups when compared with the HG group (*P* < 0.05 or *P* ≤ 0.01), among which the lowest level of intracellular enzyme activity was found in the helichrysum arenarium 25 μg/mL group (N3), as shown in Figure 1. Suggesting that the flavonoid extract of helichrysum arenarium can inhibit the extent of high glucose-induced damage in damaged myocardial cells.

### Apoptotic Protein Expression Levels in H9c2 Cells in Each Group

**Figure 2:**
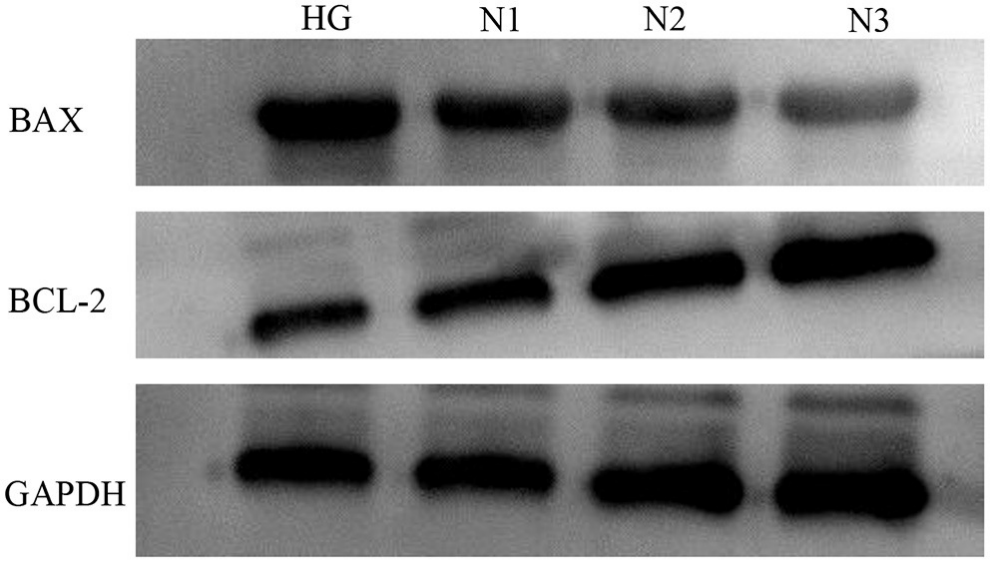
Apoptotic protein expression levels in H9c2 cells in each group. The electrophoresis of apoptosis-related proteins in each group.

Compared with the HG group, the expression of apoptosis-related factors BAX was down-regulated and Bcl-2 was up-regulated in the N1, N2, and N3 groups, and the differences were statistically significant (*P* < 0.05 or *P* ≤ 0.01), and the most significant in the helichrysum arenarium 25 μg/mL group, as shown in Figure 2. It is suggested that helichrysum arenarium can inhibit the apoptosis of damaged cardiomyocytes induced by high glucose, and the inhibition of the above-mentioned apoptosis-related analysis increased with the increase of the dose of helichrysum arenarium. The effect of apoptosis-related analysis increased with increasing dose of helichrysum arenarium.

### Expression Levels of Inflammatory Factors in H9c2 Cells in Each Group

**Figure 3:**
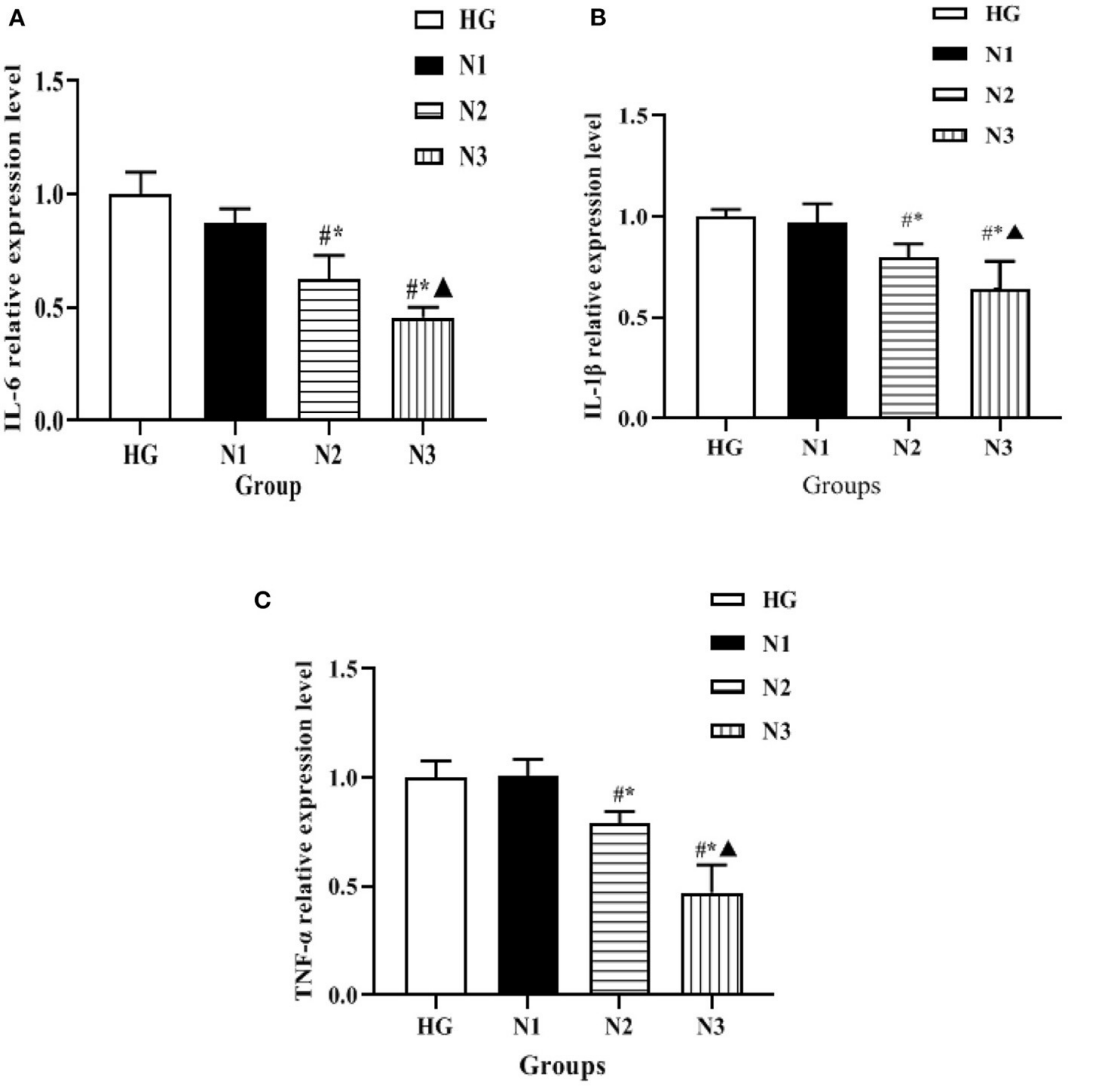
Expression levels of inflammatory factors in H9c2 cells in each group. In the above figure, **(A)** the comparison of IL-6 expression levels in the four groups of H9c2 cells, **(B)** the comparison of IL-1β expression levels in the four groups of H9c2 cells, and **(C)** the comparison of TNF-α expression levels in the four groups of H9c2 cells. Compared with HG group, ^#^*P* < 0.05; compared with N1 group,^*^P < 0.05; compared with N2 group, ^▴^*P* < 0.05.

The expression levels of mRNA of inflammatory factors such as IL-6, IL-1β, and TNF-α in the cells of each group were detected by qRT-PCR, and the changes were as follows: compared with the HG group, the mRNA levels of inflammatory factors such as IL-6, IL-1β, and TNF-α in the cells of the N2 and N3 groups were significantly lower (*P* < 0.05), and the mRNA levels of inflammatory factors such as IL-6, IL-1β, and TNF-α in the cells of the N3 group were lower than those of the N2 group (*P* < 0.05), as shown in Figure 3. Suggesting that helichrysum arenarium can inhibit the inflammatory response in damaged cardiomyocytes induced by high glucose in a dose-dependent manner.

### Body Weight, Blood Glucose, and Lipid Levels of Rats in Each Group

**Figure 4:**
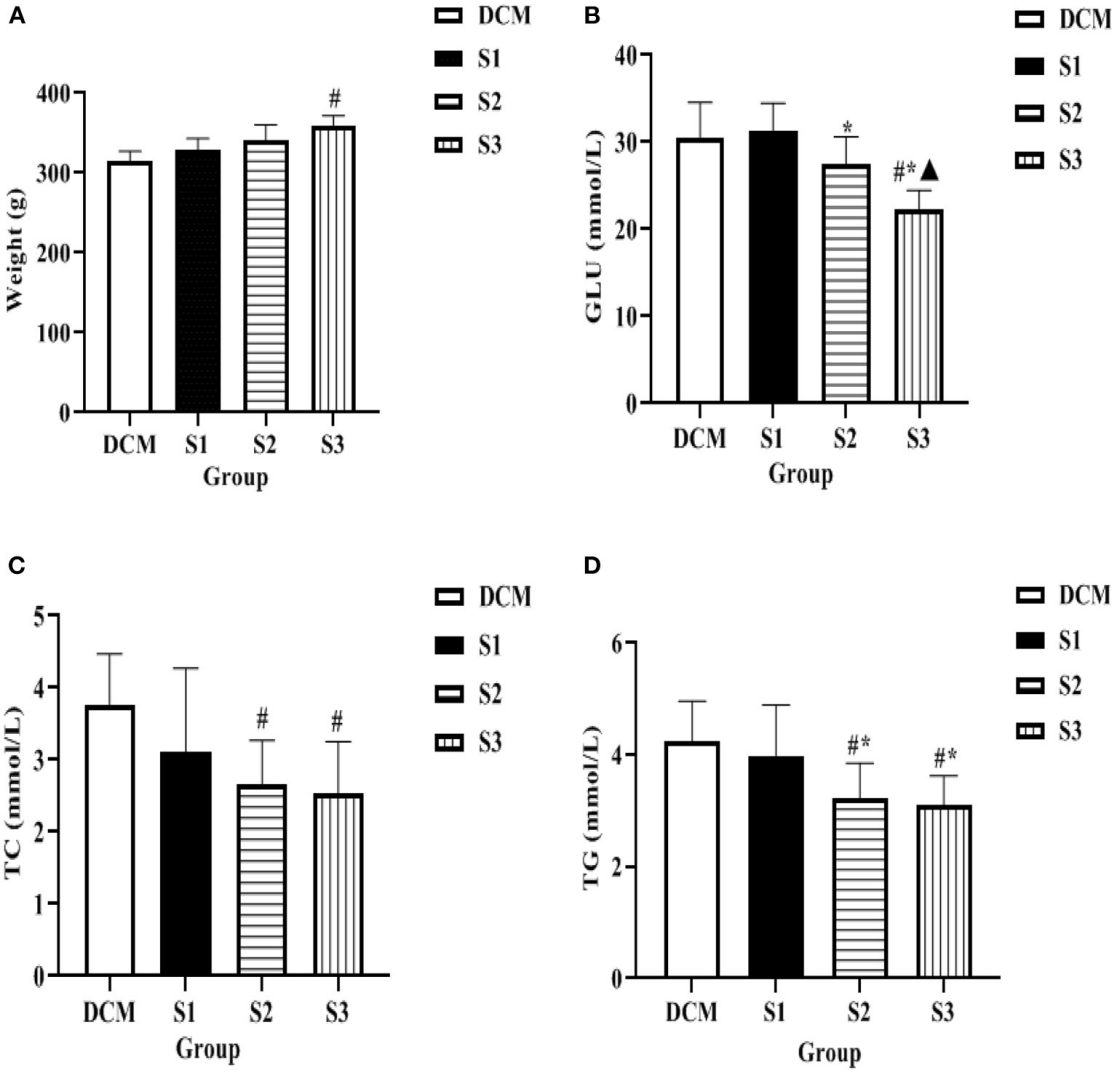
Body weight, blood glucose and lipid levels of rats in each group **(A)** is the comparison of body weight of four groups of rats, **(B)** is the comparison of GLU level of four groups of rats, **(C)** is the comparison of TC level of four groups of rats, **(D)** is the comparison of TG level of four groups of rats. Compared with the DCM group, ^#^*P* < 0.05; compared with the S1 group,^*^P < 0.05; compared with the S2 group, ^▴^*P* < 0.05.

After 10 weeks of continuous administration, the body weight of rats in S1, S2, and S3 groups gradually increased compared with the DCM group, with the most significant increase in the S3 group (*P* < 0.05 for the S3 group compared with the DCM group). Compared with the DCM group, the GLU level of S1 and S2 groups decreased, but it was not statistically significant, while the high dose of helichrysum arenarium could reduce the GLU levels in rats with DM (*P* < 0.05). At 10 weeks, the TC and TG levels of rats in S2 and S3 groups were significantly lower than those in DCM group (*P* < 0.05), as shown in Figure 4. This suggests that helichrysum arenarium can improve lipid metabolism in rats with diabetic myocarditis.

### Histomorphological Changes of Myocardium in Each Group of Rats (HE, ×400)

**Figure 5:**
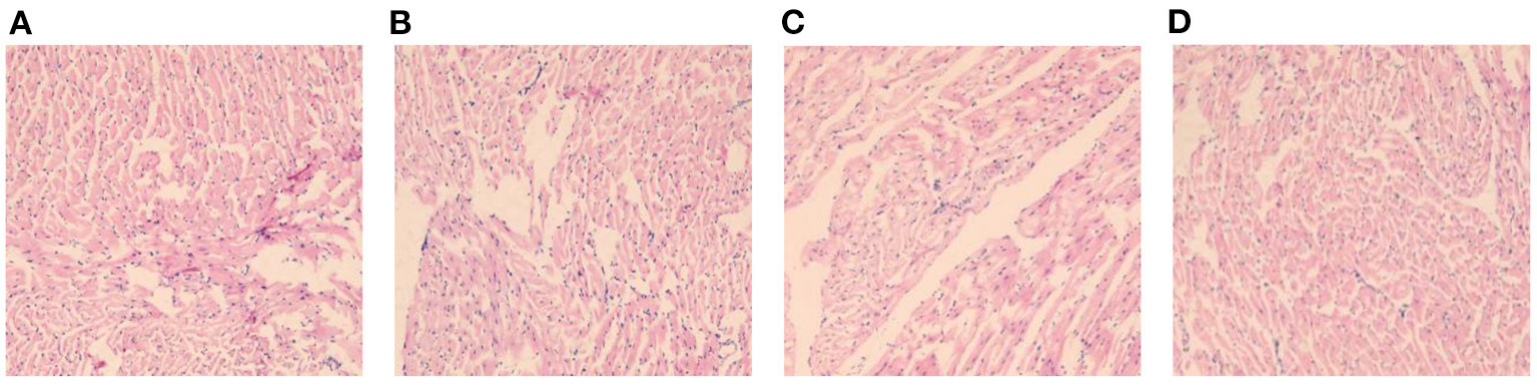
Histomorphological changes of myocardium in each group of rats (HE, ×400). **(A)** is the DCM group, **(B)** is the S1 group, **(C)** is the S2 group, and **(D)** is the S3 group.

In DCM group, myocardial fibers of mice were disordered, with uneven texture, cellular hypertrophy and deformation, increased cellular gaps, and inflammatory infiltration was seen around them. In comparison with DCM group, myocardial fibers and cells of rats were improved in S2 and S3 groups, and myocardial fibers and myocytes of S3 group were more neatly arranged and fibers were intact, and the improvement was most obvious. See Figure 5.

### Expression Levels of Inflammatory Factors in Myocardial Tissue of Rats in Each Group

**Figure 6:**
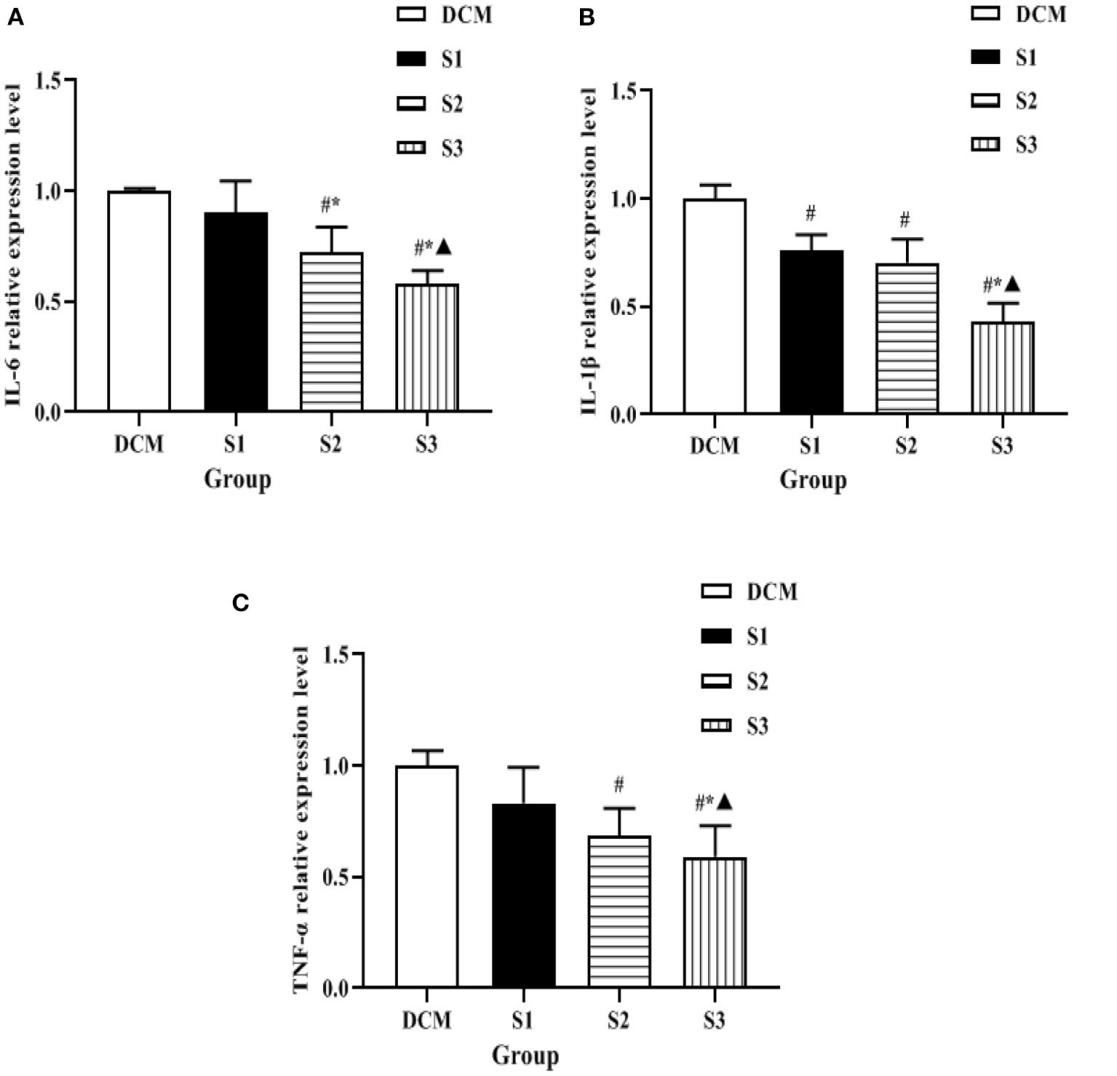
Expression levels of inflammatory factors in myocardial tissue of rats in each group. **(A)** is the comparison of IL-6 expression levels among the four groups, **(B)** is the comparison of IL-1β expression levels among the four groups, and **(C)** is the comparison of TNF-α expression levels among the four groups. Compared with the DCM group, ^#^*P* < 0.05; compared with the S1 group, ^*^P < 0.05; compared with the S2 group, ^▴^*P* < 0.05.

The expression levels of mRNA of inflammatory factors such as IL-6, IL-1β, and TNF-α in myocardial tissues of DM rat model were detected by qRT-PCR in each group, and the changes were as follows: compared with DCM group, the mRNA levels of inflammatory factors such as IL-6, IL-1β, and TNF-α in myocardial tissues of rats in S2 and S3 groups were significantly lower (*P* < 0.05); the mRNA levels of inflammatory factors such as IL-6, IL-1β, and TNF-α in myocardial tissues of rats in S3 group were lower than those in S2 group (*P* < 0.05) (see Figure 6). It was suggested that helichrysum arenarium could inhibit the myocardial inflammatory response in DM rat model and showed a dose-dependent effect.

## Discussion

The incidence of DM is increasing, and the incidence of DM-related complications is also gradually increasing due to the long-term hyperglycemic environment, irrational use of hypoglycemic drugs, and poor glycemic control after drug therapy, among which DCM is the most common (18, 19). DCM is defined as myocardial disease that occurs in patients with DM, can be excluded as being caused by other diseases, and is the leading cause of death (20). Therefore, along with aggressive glycemic control, efforts to find drugs to prevent and mitigate myocardial and vascular injury are particularly important to reduce cardiovascular events. Helichrysum arenarium has been shown to have hypoglycemic effects and potential targets for the treatment of type 2 diabetes mellitus (T2DM) have been reported in the literature (21, 22) in tissues such as liver, muscle, pancreas, and brain, and these targets provide new ideas for the study of the pharmacological mechanism of T2DM in helichrysum arenarium.

In this study, the H9c2 cardiomyoblast cell line was treated with high glucose environment to simulate a model of damaged cardiomyocytes. It was found that helichrysum arenarium could reduce the leakage of CK and LDH in cardiomyocytes induced by high glucose, and could inhibit apoptosis of damaged cardiomyocytes induced by high glucose, and the inhibitory effect was enhanced with increasing dose of helichrysum arenarium. Active enzymes such as CK and LDH present in cardiomyocytes cannot cross the cardiomyocyte membrane under normal conditions; however, when the cells are damaged, the permeability of the cell membrane increases and CK and LDH leak out of the cells. Therefore, the level of pericellular CK and LDH can reflect the degree of cardiomyocyte damage (23, 24). The Bcl-2 family is a gene family closely related to apoptosis, and the protein products of this family are functionally divided into proteins with apoptosis-promoting effects, such as Bax, and proteins with apoptosis-inhibiting effects, such as Bcl-2. Under normal physiological conditions, there is a dynamic balance between pro-apoptotic and anti-apoptotic proteins, and when the body or cell is subjected to noxious stimuli, the disruption of this balance leads to increased apoptosis (25, 26). In this study, cells cultured under high glucose environment showed a significant increase in CK and LDH content, up-regulation of intracellular BAX expression and down-regulation of Bck-2 expression over time. However, helichrysum arenarium was able to reduce CK and LDH leakage induced by high glucose, and BAX expression was down-regulated and Bck-2 expression was increased, which indicates that it has a certain ameliorative effect on myocardial injury induced by high glucose and can inhibit apoptosis of damaged myocardial cells.

The DM model was established by intraperitoneal injection of STZ, and the body weight, blood glucose and lipid levels of rats were detected after 10 weeks, and HE staining of myocardial tissues of rats was taken for pathological section. The results showed that the levels of GLU, TC and TG in S3 group were significantly lower than those in DCM group at 10 weeks. In the DCM group. HE staining in the DCM group showed that the cardiomyocytes were hypertrophied and disordered, and the intercellular space was widened. Compared with the DCM group, the structure of the cardiomyocytes in the S1, S2, and S3 groups was destroyed and the arrangement disorder was alleviated, and the S3 group improved more pronounced. Thus, it can be seen that helichrysum arenarium may improve the lipid metabolism of diabetic rats and reduce the accumulation of lipids in the myocardium, thus reducing the damage of lipids and their metabolites to the myocardium.

In recent years, with the in-depth study of clinical medicine, it was found that the subclinical inflammatory response plays an important role in the development and progression of DCM, and the inflammatory response is accompanied by the release of a large number of cytokines and acute phase proteins, such as IL-6, IL-1β, and TNF-α (27–29). The present experiment revealed that IL-6, IL-1β, and TNF-α were significantly increased in the myocardial tissue of H9c2 cells and DM rats exposed to high glucose environment, indicating that during the course of diabetic cardiomyopathy, an inflammatory response occurs in the myocardium, and this inflammatory response promotes the secretion of myocardial inflammatory factors and eventually myocardial inflammatory injury. The extract has been found to be effective in reducing the neutral fat content in the stem cells of hyperlipidemic rats and has lipid-lowering effects, in addition to its anti-atherosclerotic effects, but there are few reports on the role of helichrysum arenarium in the inflammatory response (30). In this study, we found that the expression levels of inflammatory factors such as IL-6, IL-1β, and TNF-α in the myocardial tissues of H9c2 cells and DCM rats were all decreased to different degrees, and the decrease was the most in the high-dose group. Combined with the above results, we speculate that Wax chrysanthemum may produce anti-inflammatory myocardial injury effects by reducing the expression levels of inflammatory factors such as IL-6, IL-1β, and TNF-α.

From the above analysis we are able to conclude that the helichrysum arenarium flavonoid extract can reduce the degree of damage of H9C2 cells induced by high glucose and decrease the cellular inflammatory response, and its mechanism of action may be achieved by regulating the apoptotic factors Bax and Bcl-2. In addition, the extract of helichrysum arenarium can reduce the histopathological damage of myocardium in diabetic rats, decrease the inflammatory response in the tissue, and achieve the effect of myocardial protection.

## Data Availability Statement

The original contributions presented in the study are included in the article/supplementary material, further inquiries can be directed to the corresponding author.

## Ethics Statement

The animal study was reviewed and approved by the Animal Ethics Committee of XinJiang Medical University.

## Author Contributions

HL and WL made equal contributions, intellectually, and physically. Both authors contributed to the article and approved the submitted version.

## Funding

This trial was funded by the Scientific Research Project of the Affiliated Hospital of Traditional Chinese Medicine of Xinjiang Medical University (ZYY201722) and the National Key R&D Program Project (2017YFC1703902-3).

## Conflict of Interest

The authors declare that the research was conducted in the absence of any commercial or financial relationships that could be construed as a potential conflict of interest.

## Publisher's Note

All claims expressed in this article are solely those of the authors and do not necessarily represent those of their affiliated organizations, or those of the publisher, the editors and the reviewers. Any product that may be evaluated in this article, or claim that may be made by its manufacturer, is not guaranteed or endorsed by the publisher.
